# Identification and characterization of immunomodulatory peptides from pepsin–soy protein hydrolysates

**DOI:** 10.1186/s40643-022-00526-2

**Published:** 2022-04-04

**Authors:** Lu-Sheng Hsieh, Ming-Shing Lu, Wen-Dee Chiang

**Affiliations:** Department of Food Science, College of Agriculture, Tunghai University, No. 1727, Sec. 4, Taiwan Boulevard, Xitun District, Taichung, 40704 Taiwan

**Keywords:** Immunomodulatory peptide, Isolated soy protein (ISP), Lipopolysaccharide (LPS), Molecular weight cut-off (MWCO), Pepsin, Soybean

## Abstract

To obtain immunomodulatory peptides from isolated soy protein (ISP), pepsin was selected to prepare hydrolysates and 4-h treatment (Pepsin-ISPH4h) showed the highest yield and immunomodulatory activities. The Pepsin-ISPH4h was sequentially fractionated by 30, 10 and 1-kDa molecular weight cut-off (MWCO) membranes, in which 1-kDa MWCO permeate (1P) exhibited the most significant enhancement of phagocytosis activity without causing excessive inflammation as compared with Pepsin-ISPH4h. To further purify and enhance the immunomodulatory activity, 1P was distinct by high-performance liquid chromatography equipped with a reverse-phase column and in vivo immunomodulatory activity of fractions was examined in mice. Fraction 1 (F1) significantly elevated phagocytosis activity of mice spleen macrophages and neutrophils. However, increase of phagocytosis activity did not result from the induction of macrophages M1 or M2 polarization. The immunomodulatory peptide sequence, EKPQQQSSRRGS, from F1 was identified by LC–MS/MS. Phagocytosis activity and macrophage M1 polarization were elevated by synthetic peptide treatment. Hence, our results indicated that isolated soy protein hydrolysates prepared by pepsin could provide a source of peptides with immunomodulatory effects.

**Graphical Abstract:**

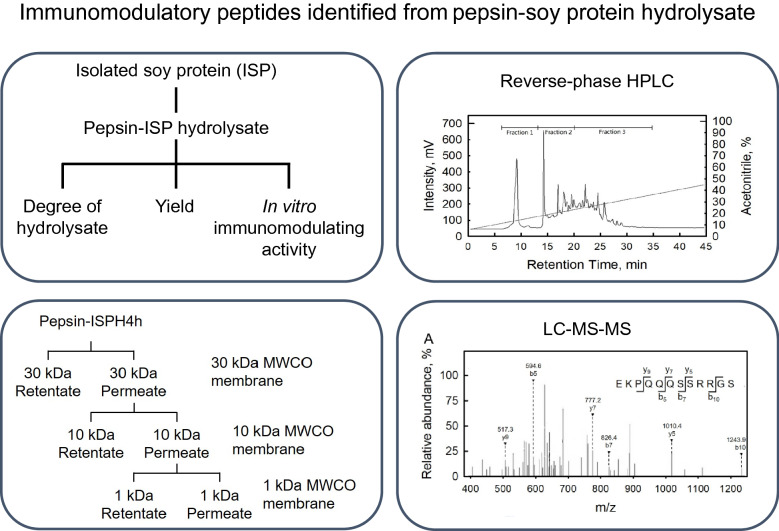

## Introduction

Human immunity is influenced by many factors such as age, dietary habit, exercise, stress, and so on (Hamer et al. 2012; Marques et al. 2015). Busy life style causes imbalanced immune modulations in people of modern world; therefore, maintaining normal daily life style as well as supplementing immune modulators can rectify immune imbalance (Santiago-López et al. 2016; Yu et al. 2018; Polak et al. 2021). The studies of protein enzymatic hydrolysates are initially focused on improving dietary and nutritional functions (Adler-Nissen 1986), for example human gut intestinal systems absorb small peptides (dipeptide or tripeptide) better than free amino acids (Ziegler et al. 1990; Siemensma et al. 1993; Bueno-Gavilá et al. 2021; Zaky et al. 2022). Recently, immunomodulatory peptides showed beneficial effects on human health are prepared from food-based proteins such as chum salmon (Yang et al. 2009), Alasaka pollock (Hou et al. 2012), rohu egg (Chalamaiah et al. 2014, 2018), wheat germ globulin (Wu et al. 2017), rice (Fang et al. 2019), false starwort (*Pseudostellaria heterophylla*; Yang et al. 2020), duck egg ovalbumin (He et al. 2021), and *Stevia rebaudiana* (Li et al. 2021), implying that an increasing number of scientists are attracted and devoted into this research field.

The primary function of mammalian immune system is to prevent contagious illnesses by building up a complicate firewall by cells and proteins (Bayne 2013), and can be divided into innate and adaptive immune systems (Iwasaki and Medzhitov 2015). Macrophages and neutrophils are endocytic defense cells of innate immune system that can nonspecifically engulf external pathogens and trigger inflammation responses by releasing NO and cytokines (Gordon 2016). Proper inflammation response can help human body to defense invasions of pathogens; however, hyperinflammation may cause tissues damages (Ginderachter et al. 2006). Cytokines, such as interleukin-6 (IL-6), interleukin-10 (IL-10), and tumor necrosis factor-α (TNF-α), are tiny proteins, modulating the growth and functions of immune cells (Kim et al. 2008; Ahn et al. 2015). Macrophage cells can further polarize into M1 and M2 types, in which M1 cells can produce high amount of pro-inflammatory cytokines and reactive oxygen species to promote inflammation, whereas M2 cells secrete anti-inflammatory cytokine, IL-10, to repair damaged tissues (Murray et al. 2014).

Soybean (*Glycine max* L.) protein is an important source with properties of high yield, low price, high nutritive value, and broad applications (Ricker et al. 2004; Coscueta et al. 2019; Akbarian et al. 2022). Peptides identified from soy protein hydrolysates have shown functions of anti-oxidation (Ranamukhaarachchi et al. 2013; Ashaolu 2020), stimulating lipolysis (Tsou et al. 2012), anti-angiotensin I-converting enzyme activity (Rho et al. 2009), and immunomodulatory effects (Egusa and Otani 2009; Dia et al. 2014; Zhang et al. 2021). The goal of this study was to isolate and purify immunomodulatory peptides from pepsin-isolated soy protein hydrolysates (Pepsin-ISPH) using a combination of molecular weight cut-off module and reverse-phase high-performance liquid chromatography. Peptides were further identified by mass spectrometry and synthetic peptides were used for investigating its mechanisms of immunomodulation functions.

## Materials and methods

### Materials and chemical reagents

Isolated soy protein (ISP) and NEW Soy 88 were purchased from Gemfont Co., Taipei, Taiwan. Pepsin from porcine gastric mucosa, sodium dodecyl sulfate (SDS), *o*-phthalaldehyde (OPA), Leu-Gly dipeptide, lipopolysaccharide (LPS), dimethyl sulfoxide (DMSO), sodium carbonate (NaHCO_3_), and 3-(4,5-dimethylthiazol-2-yl)-2,5-diphenyltetrazolium bromide (MTT) were obtained from MilliporeSigma, Darmstadt, Germany. Dulbecco’s modified Eagle medium (DMEM) was purchased from Gibco, TX, USA. l-Glutamate and charcoal/dextran-treated fetal bovine serum (FBS) were obtained from Biological Industries, CT, USA. Molecular weight cut-off (MWCO) membranes, ER 30 kDa, PW 10 kDa, and GE 1 kDa, were obtained from Osmonics Inc., MN, USA. All chemical reagents used were American Chemistry Society (ACS) grade or better.

### Preparation of enzymatic hydrolysate, measurement of hydrolysis ratio, and determination of soluble nitrogen by Kjeldahl method

The 2.5% (w/v) ISP was dissolved in 0.2 M phosphate buffer (pH 2.0) and digested by pepsin (S:E ratio = 100:1) at 37 °C. Hydrolysates of isolated soy protein (ISPH) were collected at 0, 0.5, 1, 2, 4, and 6 h, then pepsin was inactivated by boiling for 15 min followed by storing at − 20 °C until use.

The degree of hydrolysis (DH) was measured by OPA method using the dipeptide, Leu-Gly, as standard (Nielson et al. 2001). DH (%) was indicated as:

$$\mathrm{DH }\left(\mathrm{\%}\right)=\frac{{H}_{\mathrm{sample}}}{{H}_{\mathrm{total}}} \times 100\%.$$

*H*_sample_ represents α-amino group concentration (mmol/mL); *H*_total_ represents total peptide number of ISPH (7.8 mEq α-amino group/g).

The soluble nitrogen of hydrolysate was prepared by adding 20% (w/v) trichloroacetic acid, and its nitrogen content was estimated by Kjeldahl method according to Tsou et al. (2012). Yield (*N* mg/mL) was indicated as:

$$\mathrm{Yield }\left(N\mathrm{ mg}/\mathrm{mL}\right)=\frac{\left(V1-V2\right) \times C \times 14}{\mathrm{Sample volume }(\mathrm{mL})}.$$

*V*1: titration volume of sample (mL); *V*2: titration volume of blank (mL); *C*: concentration of HCl (0.1 N × titer); 14: atomic mass of nitrogen.

### Fractionation of hydrolysate by molecular weight cut-off

Hydrolysate of 4-h pepsin-treated isolated soy protein (Pepsin-ISPH4h) was sequentially fractionated by a membrane MWCO module with 30 kDa, 10 kDa, and 1 kDa to obtain retentates and permeates. One volume of Pepsin-ISPH4h was initially filtered by a 30-kDa MWCO membrane to acquire 1:9 ratio of retentates (30R) and permeates. The 30-kDa permeate was then filtered by a 10-kDa MWCO membrane to acquire 1:9 ratio of retentates (10R) and permeates. The 10-kDa permeate was then filtered by a 1-kDa MWCO membrane to acquire 1:9 ratio of retentates (1R) and permeates (1P).

### Fractionation of 1P fraction by reverse-phase HPLC

Fraction 1P was further fractionated using a reverse-phase high-performance liquid chromatography applied on an InertSustain^®^ C_18_ column (10 × 250 mm, 5 μm, GL Sciences, Japan) with a linear gradient of acetonitrile from 0 to 45% in 45 min at a flow rate of 2 mL/min. The elution signals were monitored at 214 nm.

### Culture of mouse macrophage cells

Mouse macrophage RAW264.7 cell line was obtained from Bioresource Collection and Research Center (BCRC 60001), Hsinchu, Taiwan. Cells were grown in DMEM medium supplemented with 10% FBS, 1.6 g/L NaHCO_3_, and 2 mM l-glutamine, maintaining in a 5% CO_2_ incubator at 37 °C. Cells were subcultured every 48–72 h, and discarded after 50 generations.

### Cell viability—MTT test

The cytotoxicity activity against RAW264.7 macrophage was investigated by MTT assay (Mosmann et al. 1983; Tsou et al. 2012). Cells were cultured in a 96-well microtiter plate (1 × 10^5^ cells/well) for 24 h followed by incubating with various ISPH for 24 h. Cells were washed with phosphate buffer saline (PBS) and then incubated with MTT solution (0.5 mg/mL) at 37 °C for 4 h. Methanol treatment was used as negative control. DMSO solution was applied to resuspend the MTT formazan for 20 min. The absorbance was determined at 595 nm using a microtiter plate reader (BioTek, VT, USA). Cell viability (%) was indicated as:

$$\mathrm{Cell viability }\left(\mathrm{\%}\right)=\frac{{({\mathrm{OD}}_{\mathrm{sample}}-\mathrm{OD}}_{\mathrm{methanol}})}{{({\mathrm{OD}}_{\mathrm{control}}-\mathrm{OD}}_{\mathrm{methanol}})} \times 100\%.$$

OD_sample_: absorbance at 595 nm of sample; OD_control_: absorbance at 595 nm of untreated sample as control; OD_methanol_: absorbance at 595 nm of sample treated with methanol as negative control.

### Determination of nitrogen oxide production

The NO production was measured by Griess assay (Ahn et al. 2015). RAW264.7 macrophage cells were cultured in a 96-well microtiter plate (1 × 10^5^ cells/well) for 24 h followed by incubated with 1 ppm LPS and/or various ISPH for 24 h. After treatment, 50 μL culture medium was mixed with 50 μL Griess reagent and then incubated in dark place for 10 min. The absorbance of the mixture was determined at 550 nm using a Microtiter plate reader (BioTek, VT, USA). The concentration of NO was calculated using a standard curve generated from sodium nitrite dissolved in DMEM medium.

### Phagocytosis assay

RAW264.7 macrophage cells were cultured in a 96-well microtiter plate (1 × 10^5^ cells/well) for 24 h followed by incubated with various ISPH for 24 h. One ppm LPS treatment was used as positive control. *E. coli* BL21 cells transformed with pEGFP plasmid was added in a 96-well microtiter plate (5 × 10^6^ cells/well) and centrifuged at 120*g* at 4 °C for 5 min to precipitate *E. coli* cells to be phagocytosed by macrophage for 2 h. Trypan blue regent (2 ×) was added in a 96-well microtiter plate and incubated for 2 min. Trypan blue was removed and fluorescence signal was measured with excitation wavelength at 485 nm and emission wavelength at 538 nm. Relative phagocytosis was indicated as:

$$\mathrm{Relative phagocytosis }\left(\mathrm{\%}\right)=\frac{{\mathrm{OD}}_{\mathrm{sample}}}{{\mathrm{OD}}_{\mathrm{LPS}}} \times 100\%.$$

### Determination of pro-inflammatory cytokines in RAW246.7 macrophage cells stimulated by LPS

The levels of interleukin-6 (IL-6) and interleukin-10 (IL-10) were measured by mouse IL-6 and IL-10 Quantikine ELISA kits (R&D systems, MN, USA) following to the manufacturer’s instruction. RAW264.7 macrophage cells were cultured in a 96-well microtiter plate (1 × 10^5^ cells/well) for 24 h and culture media were stored at − 20 °C until use. Capture antibodies were coated onto 96-well microtiter plate at 4 °C overnight. After blocking, microtiter plate was washed three times and 100 μL/well standards or culture media were added and incubated at room temperature for 2 h. Microtiter plate was washed three times and then 100 μL/well detection antibodies were added and incubated at room temperature for 2 h. Microtiter plate was washed three times and then 100 μL/well streptavidin–HRP was added and incubated in dark for 20 min. Plate was washed three times and then 100 μL/well substrate solution was added and incubated in dark condition for 20 min. After incubation, 50 μL stop solution was added and the absorbance of the mixture was determined at 450 nm using a microtiter plate reader (BioTek, VT, USA). The concentrations of cytokinins were calculated using a standard curve generated from various concentrations of standards.

### Mouse spleen cell endocytosis assay

Male C57BL/6J mouse (19 weeks) was injected intraperitoneally with 5 mg/kg samples, and killed three days after injection. Spleen was collected and placed in 5 mL YAC medium (RPMI 1640, 2.2 g/L NaHCO_3_, 2 mM l-glutamine, 10% FBS) and then ground with 200 mesh sieve. Supernatant was removed by centrifugation at 300*g*, 4 °C, for 10 min. Pellet/cell was kept and resuspended in 5 mL 0.1 × Hank’s balanced salt solution (HBSS) to disrupt red blood cells and then added 10 mL HBSS. Supernatant was removed by centrifugation at 300*g*, 4 °C, for 10 min, and pellet/cell was resuspended in YAC medium and adjusted to 2 × 10^7^ cells/mL by a FACSCalibur™ Flow Cytometer (BD Bioscience, NJ, USA). Spleen lymphocyte cells were cultured in a 96-well microtiter plate (1 × 10^5^ cells/well) and BioParticles^®^ FITC–*Escherichia coli* (2.5 × 10^6^ cells/well) was added and incubated at 37 °C for 2 h. After removing supernatant, lymphocyte cells were mixed with 100 μL trypan blue and fluorescence signal was measured by FACSCalibur™ Flow Cytometer (BD Bioscience, NJ, USA). Positive fluorescence level was indicated as:

$$\mathrm{positive fluorescence level}=M1+M2\times 10+M3\times 100+M4\times 1000.$$

*M*1: percentage cell within 10^1^ fluorescence signal; *M*2: percentage cell within 10^2^ fluorescence signal; *M*3: percentage cell within 10^3^ fluorescence signal; *M*4: percentage cell within 10^4^ fluorescence signal.

### Differentiation of M1 and M2 macrophages

Mouse spleen lymphocyte cells were harvested as mentioned previously. Surface biomarkers, PE-anti-mouse CD68, PE/Cy5-anti-mouse CD197, and FITC-anti-mouse CD206 antibodies, were obtained from Biolegend, CA, USA. Nine μL fluorescence conjugated antibodies were mixed with 50 μL cell suspensions and incubated at 4 °C in dark for 30 min. Cells were washed and resuspended in 200 μL analysis buffer (RPMI 1640, 5% FBS, 2 mM l-glutamate, and 2 × Dulbecco’s phosphate buffer saline). Two differentiated cells, M1 (CD68^+^/CD197^+^) and M2 (CD68^+^/CD206^+^) were analyzed using a FACSCalibur™ Flow Cytometer (BD Bioscience, NJ, USA).

### Identification of anti-inflammatory peptide by LC–MS/MS and peptide synthesis

Amino acid sequence identification of anti-inflammation peptide was analyzed by UPLC (UltiMate 3000, ThermoFisher, MA, USA) followed by quadruple tandem ion trap mass spectrometer (Q-TOF/MS/MS; Bruker micrOTOF-Q III, Bruker Daltonic, Germany) equipped with an electrospray ionization source in Center of Precision Instrument, Tunghai University, Taichung, Taiwan. Immunomodulatory peptides isolated from pepsin–soybean hydrolysate were chemically synthesized by Yao Hong Biotechnology Inc., New Taipei City, Taiwan. Peptides, 5 mg/kg and 25 mg/kg, were used in endocytosis assay and macrophage phenotype assay, respectively.

### Statistical analysis

Results were expressed as mean ± standard deviation (SD) and analyzed using Statistical Analysis System (SAS/STAT^®^ software, NC, USA). Mean with different letters were labeled as significantly different (*p* < 0.05) by Duncan’s multiple range test.

## Results and discussion

### Effect of pepsin hydrolysis of ISP on phagocytosis, NO formation, and cell viability in RAW264.7 macrophage cells

**Fig. 1:**
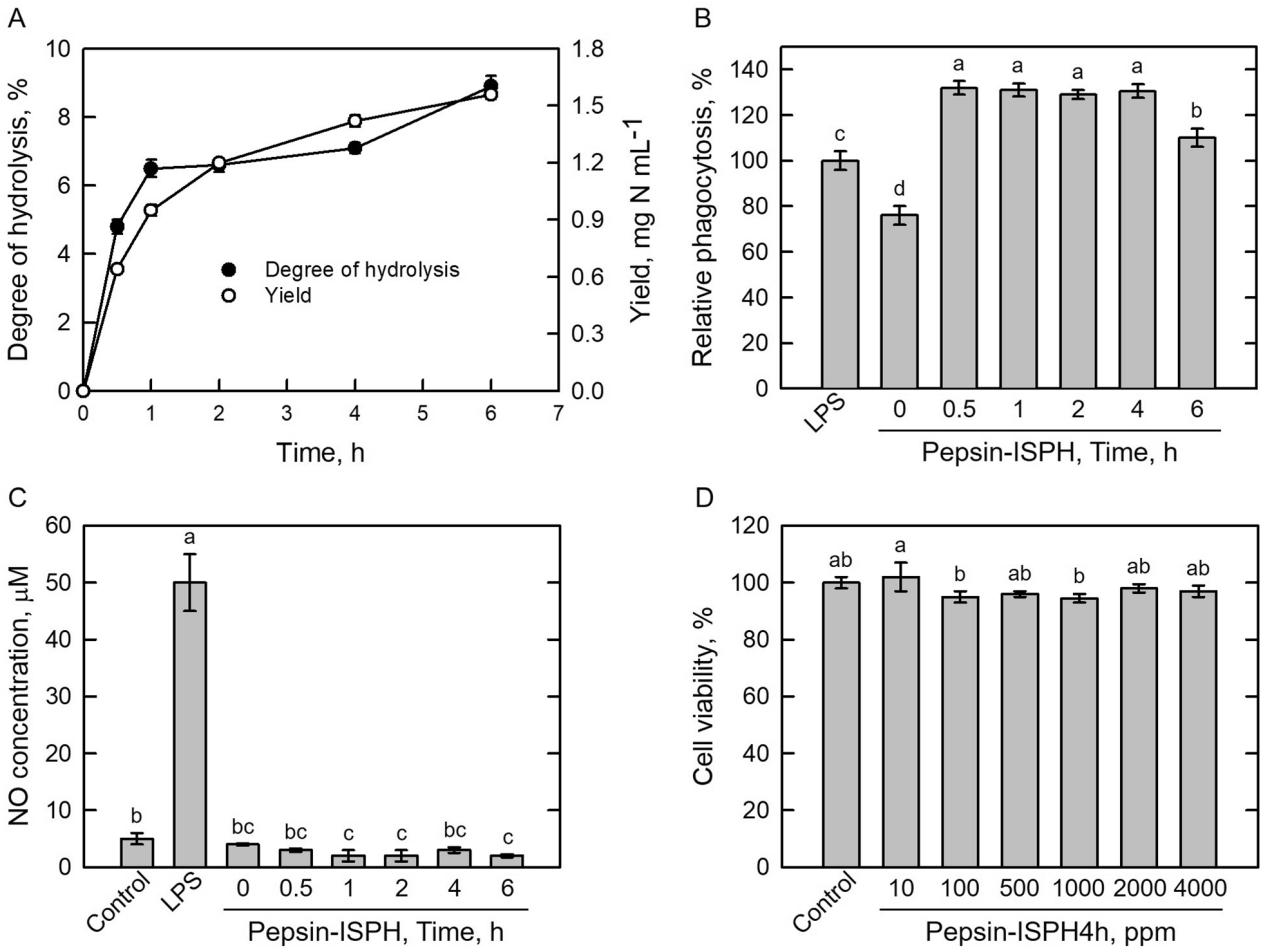
Preparation of pepsin-isolated soy protein hydrolysate (Pepsin-ISPH). **A** effect of different hydrolysis time of isolated soy protein hydrolysate by pepsin on degree of hydrolysis (●) and yield (○) in terms of TCA soluble nitrogen. **B** effect of 4000 ppm Pepsin-ISPH with different hydrolysis time on relative phagocytosis (%) in mouse RAW246.7 macrophages. **C** effect of 4000 ppm Pepsin-ISPH with different hydrolysis time on relative phagocytosis (%) in mouse RAW246.7 macrophages. **D** dosage effect of Pepsin-ISPH4h on cell viability (%) in mouse RAW246.7 macrophages. LPS (1 ppm) was used as positive control. Bars represent mean ± standard deviation (SD; *n* = 3). Mean with different letters are significantly different (*p* < 0.05) by Duncan’s multiple range test

Degree of enzymatic hydrolysis has been shown to influence functions of peptides (Jamdar et al. 2010; Liu et al. 2010; Tsou et al. 2012). To investigate immunomodulatory effects of soy protein hydrolysate, hydrolysates (Pepsin-ISPH) were obtained with the degree of hydrolysis (DH, %) of 4.8%, 6.5%, 6.6%, 7.1%, and 8.9% together with yield of 0.64, 0.95, 1.2, 1.42, and 1.56 mg nitrogen per mL, respectively (Fig. 1A). The DH of Pepsin-ISPH was positively correlated to yield in peptic hydrolysis time; the result was similar to previous studies (Jamdar et al. 2010; Liu et al. 2010; Tsou et al. 2012; Toopcham et al. 2017). In terms of phagocytosis activity, Pepsin-ISPH from 0.5 to 4 h, were 1.3-fold higher than that of LPS-treated RAW264.7 macrophage cells, whereas Pepsin-ISPH6h only showed slightly 1.1-fold increase (Fig. 1B). NO formation (Fig. 1C) and cell viability (Fig. 1D) were monitored in LPS and Pepsin-ISPH-treated RAW264.7 macrophage cells. NO was significantly produced in LPS-treated RAW264.7 macrophage cells (Dia et al. 2014; Ahn et al. 2015; Li et al. 2021); however, significant effects were not observed in pepsin hydrolysates-treated RAW264.7 macrophage cells (Fig. 1C). According to MTT assay on cell viability (Fig. 1D), Pepsin-ISPH in the ranges from 10 to 4000 ppm was not toxic to RAW264.7 macrophage cells (Fang et al. 2019; Lee et al. 2021). Next, hydrolysate of isolated soy protein digested with pepsin for 4 h (Pepsin-ISPH4h) was used to further fractionation to enrich the ability of immunomodulation.

### Effect of pepsin-ISPH separated by different MWCO membranes on phagocytosis, NO formation, and cell viability in RAW264.7 macrophage cells

**Fig. 2:**
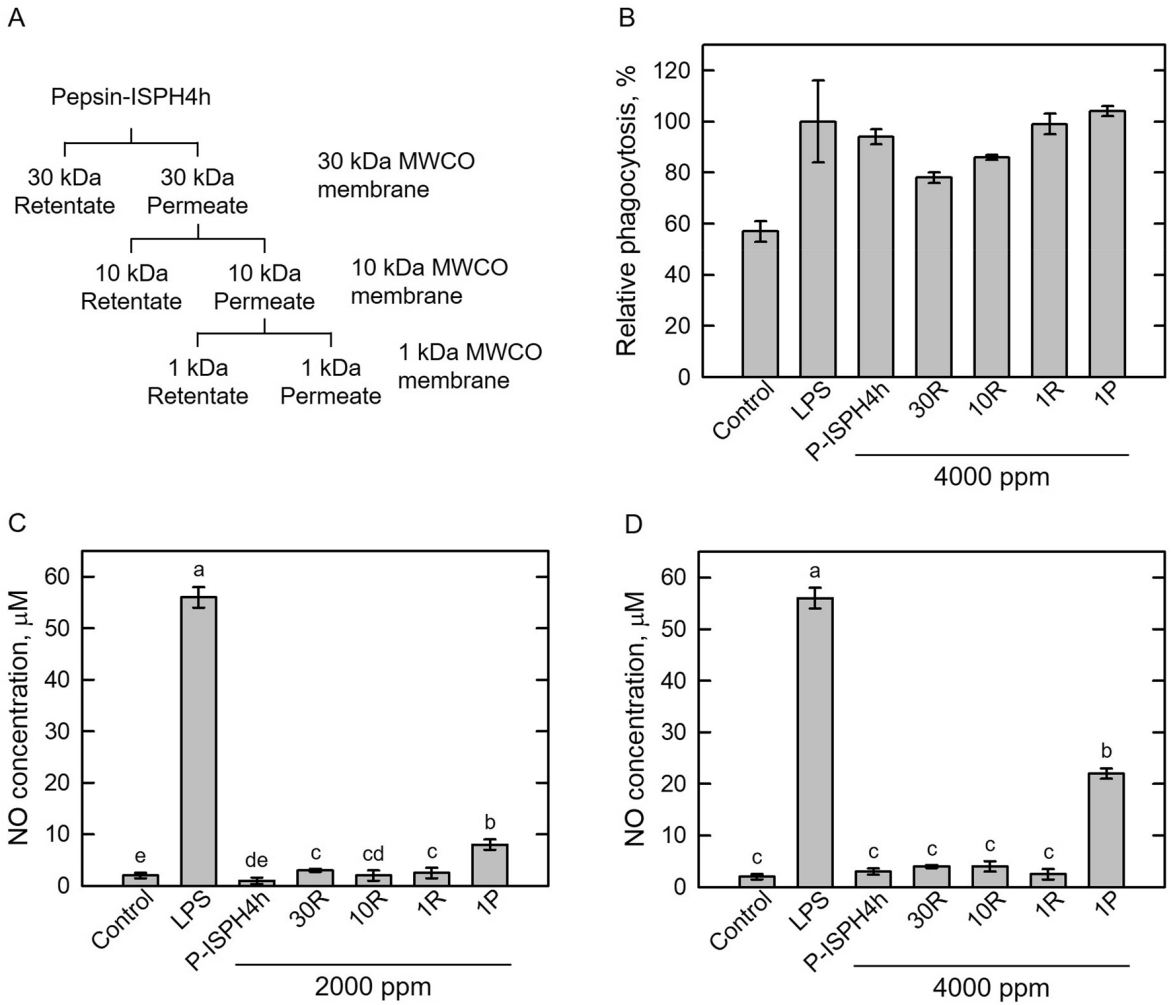
Fractionation of Pepsin-ISPH by molecular weight cut-off (MWCO) membrane. **A** fraction chart. **B** effect of 4000 ppm Pepsin-ISPH and its retentates/permeate on relative phagocytosis (%) in mouse macrophage RAW246.7 cells. Dosage effect of Pepsin-ISPH and its retentates/permeate, 2000 ppm (**C**) and 4000 ppm (**D**) on NO concentration in mouse macrophage RAW246.7 cells. LPS (1 ppm) was used as positive control. Bars represent mean ± standard deviation (SD; *n* = 3). Mean with different letters are significantly different (*p* < 0.05) by Duncan’s multiple range test

Pepsin-ISPH4h was sequentially fractionated using a membrane MWCO module with three different molecular weights, 30 kDa, 10 kDa, and 1 kDa, to acquire three retentates and one permeate fractions, namely 30R, 10R, 1R, and 1P (Fig. 2A), for further enhancing its immunomodulatory activity (Tsou et al. 2012). Next, the effects of phagocytosis of mouse macrophage cells were examined (Fig. 2B). The phagocytosis ability of the 4000 ppm 1P-treated RAW264.7 macrophage cells was slightly higher than that of LPS-treated control cells, suggesting that peptides in the 1P fraction can boost the function of RAW264.7 macrophage cells. To examine the dosage effects on NO formation, RAW264.7 macrophage cells were treated with 2000 ppm (Fig. 2C) or 4000 ppm (Fig. 2D) hydrolysate, and LPS treatment was used as positive control. Likewise, NO was significantly produced in LPS-treated RAW264.7 macrophage cells (Fig. 2C, D). NO formation by 1P treatment was increased in a dosage-dependent manner (Fig. 2C, D), indicating that peptide(s) in 1P fraction can induce NO formation in RAW264.7 macrophage cells. Previous studies had shown that proper NO formation can help immune system and macrophages to destroy tumor cells as well as invasive pathogens (Zheng et al. 2014; Fang et al. 2019; Li et al. 2021); moreover, NO formation in over 2000 ppm dosage of the 1P fraction-treated macrophages were not correlated to negative inflammation reaction (Fig. 2C). As a result, the 1P fraction was used for further fractionation to enrich the ability of immunomodulation effects.

### Effect of 1-kDa permeate (1P) on phagocytosis, NO formation, cell viability, and pro-inflammatory cytokines production in RAW264.7 macrophage cells

**Fig. 3:**
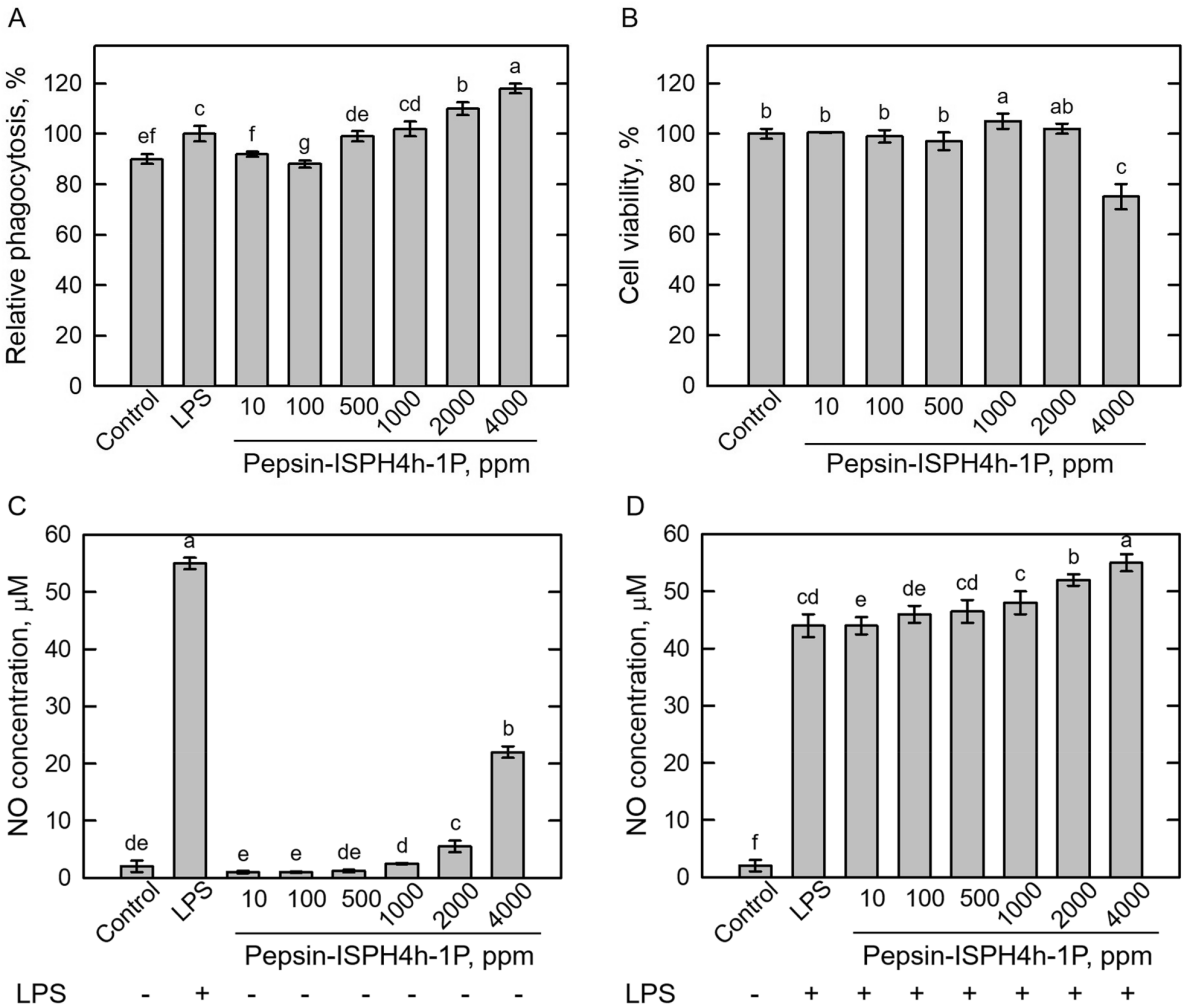
Determination of anti-inflammatory activity of Pepsin-ISPH4h-1P fraction. Dosage effect of Pepsin-ISPH4h-1P on relative phagocytosis (%, **A**), cell viability (%, **B**), and NO formation in the absence (**C**) or presence (**D**) of 1 ppm LPS in mouse macrophage RAW246.7 cells. Bars represent mean ± standard deviation (SD; *n* = 3). Mean with different letters are significantly different (*p* < 0.05) by Duncan’s multiple range test

To investigate the immunomodulation function of the 1-kDa permeate (1P), phagocytosis activity (Fig. 3A), cell viability (Fig. 3B) and NO formation in the absence (Fig. 3C) or presence (Fig. 3D) of LPS were used to estimate the optimum working concentration of the 1P fraction. In Fig. 3A, the maximum phagocytosis activity was monitored in the 4000 ppm 1P-treated RAW264.7 macrophage cells. Except 4000 ppm 1P treatment, cell viabilities were not influenced by 1P treatments from 10 to 2000 ppm (Fig. 3B). Furthermore, 4000 ppm 1P treatment led to remarkable NO formations with or without LPS (Fig. 3C, D).

**Fig. 4:**
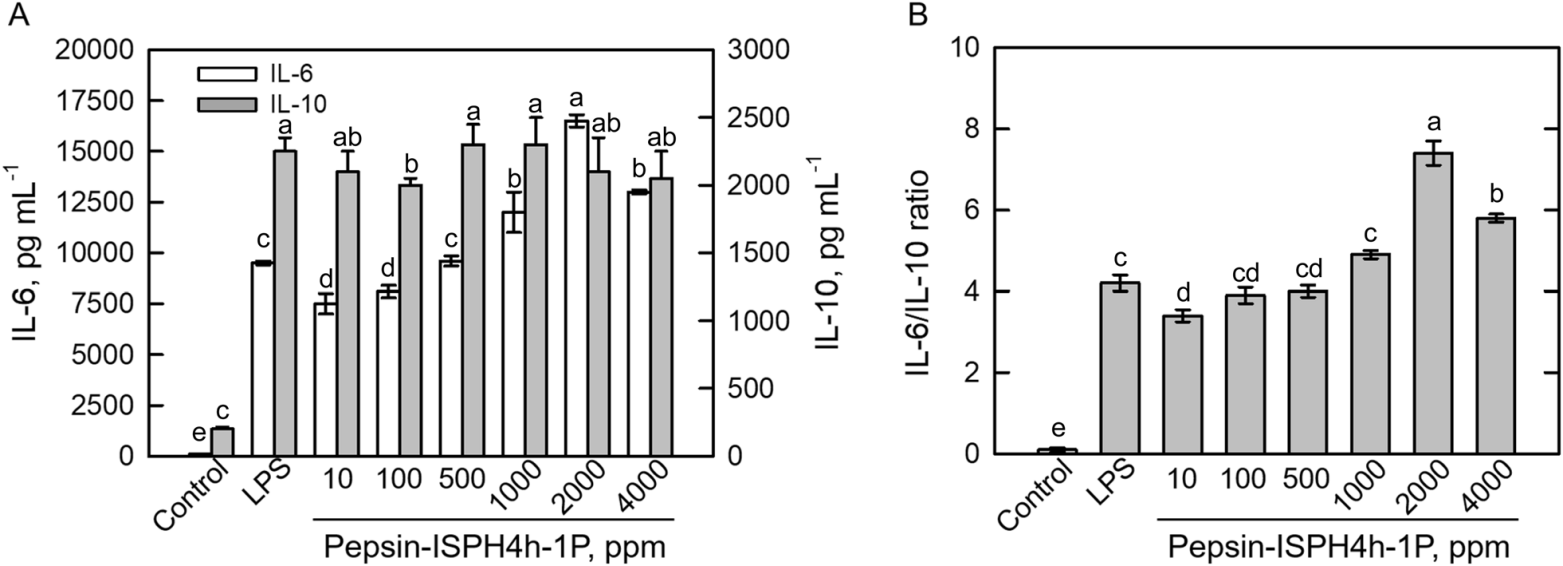
Determination of pro-inflammatory cytokines in LPS–Pepsin-ISP4h-1P-costimulated RAW264.7 macrophage cells. **A** dosage effect of Pepsin-ISPH4h-1P with 1 ppm LPS co-stimulation on IL-6 (gray column) and IL-10 (white column) productions. **B** dosage effect of Pepsin-ISPH4h-1P with 1 ppm LPS co-stimulation on IL-6/IL-10 ratio. Bars represent mean ± standard deviation (SD; *n* = 3). Mean with different letters are significantly different (*p* < 0.05) by Duncan’s multiple range test

Interleukin-6 (IL-6) and interleukin-10 (IL-10) are major pro-inflammatory cytokines production in macrophage cells (Ahn et al. 2015; Toopcham et al. 2017; Lee et al. 2021; Li et al. 2021). During inflammation response, cells can release a large amount of IL-6 to promote the formation of NO as well as to activate phagocytosis of macrophage cells (Minato and Abe 2013; Li et al. 2021), whereas IL-10 exhibits anti-inflammatory ability to reduce NO formation as well as to decrease inflammatory cytokinins secretions (Asadullah et al. 2003; Lee et al. 2021). To study the effect of the 1P fraction on cytokine production, RAW264.7 macrophage cells were treated with various dosages of the 1P fraction, and LPS treatment was used as positive control. The formation of IL-6 was intercorrelated to the increased concentrations of the 1P fraction (Fig. 4A); however, the amount of IL-10 was constantly induced by 1P treatments which was independent on its dosages (Fig. 4A). In addition, IL-6/IL-10 ratio was compared in LPS-treated cells and the maximum ratio was monitored in the 2000 ppm 1P-treated macrophage cells (Fig. 4B). The higher IL-6/IL-10 ratio indicated that cells were prone to inflammatory response (Song et al. 2016; Sapan et al. 2017; Koyama et al. 2021). As a result, 1P treatment cannot trigger severe inflammation response and was had no inhibitory effect on LPS-induced inflammatory effects. Also, similar results were reported in shark derived protein hydrolysate (Mallet et al. 2014) and rice proteins (Wen et al. 2021).

### Effect of 1P fractions, F1–F3, on phagocytosis and polarization in RAW264.7 macrophage cells

**Fig. 5:**
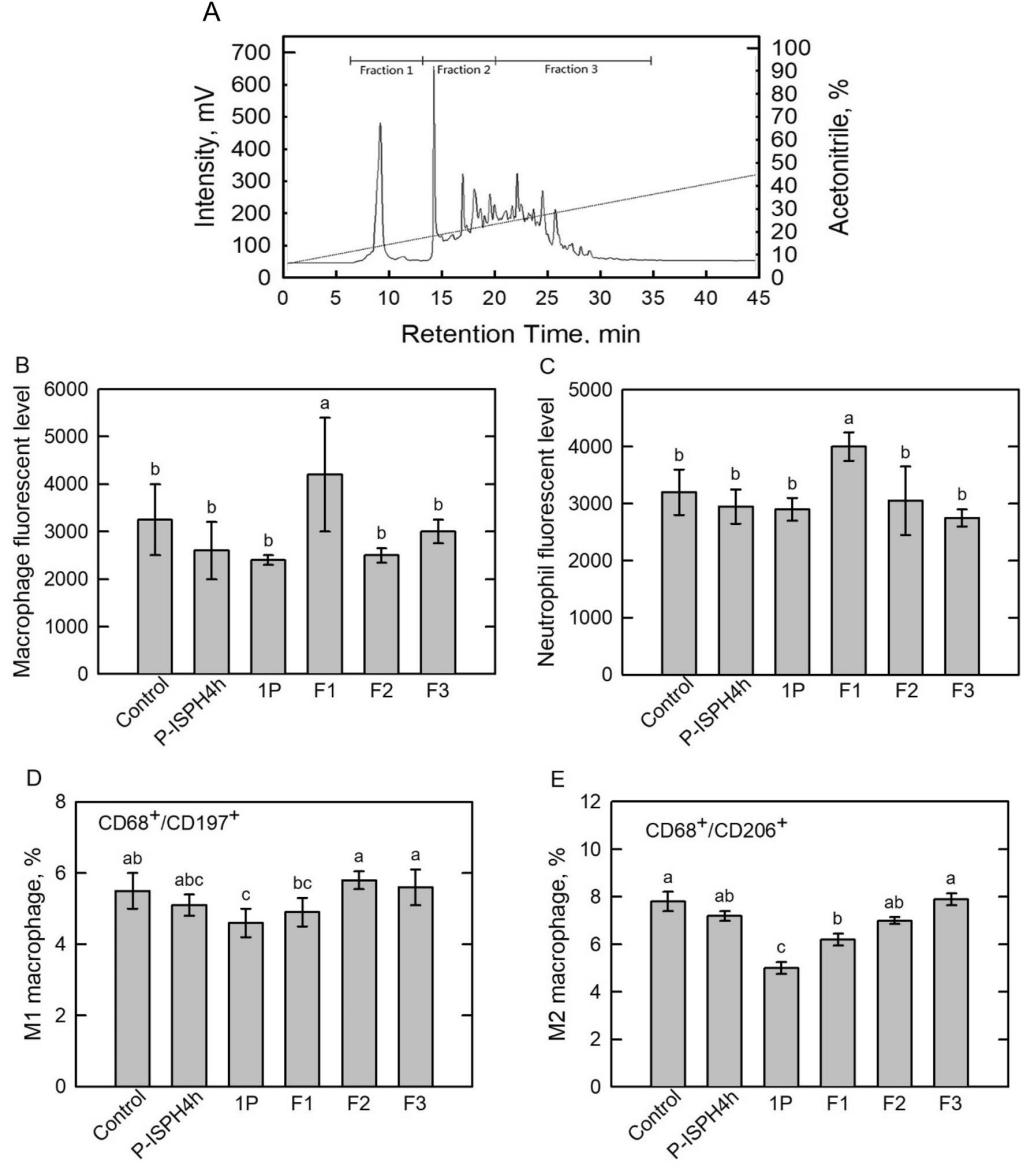
Determation of anti-inflammatory activity of Pepsin-ISPH4h-1P fractions. **A** Pepsin-ISPH-4 h-1P was fractionated into F1–F3 using a reverse-phase high-performance liquid chromatography. Effects of Pepsin-ISPH4h, Pepsin-ISP4h-1P, and F1–F3 fractions on positive fluorescent level in macrophages (**B**), neutrophil (**C**), M1 (**D**) or M2 (**E**) phenotype polarization in mouse spleen in vivo. Bars represent mean ± standard deviation (SD; *n* = 3). Mean with different letters are significantly different (*p* < 0.05) by Duncan’s multiple range test

Fraction 1P exhibited the immunomodulatory ability to promote cell phagocytosis was further separated by reverse-phase HPLC into three fractions (Fig. 5A). Based on its retention time, Fraction 1 (F1), Fraction 2 (F2), and Fraction 3 (F3) were in between 6 and 14 min, 14–21 min, and 21–35 min, respectively (Fig. 5A). For endocytosis activity assay, macrophage (Fig. 5B) and neutrophil cells (Fig. 5C) were harvested from mice spleens and then incubated with transgenic recombinant green fluorescence protein (GFP) produced *E. coli* cells (Gille et al. 2006). The fluorescent level was represented as the endocytosis activity (Jiang et al. 2007). As shown in Fig. 5C, F1 fraction significantly increased endocytosis activity in macrophage cells, whereas F2 and F3 fractions exhibited no influences as control. In addition, F1 treatment also enhanced endocytosis activity of neutrophil cells (Fig. 5D). Next, Pepsin-ISPH4h, 1P, and F1–F3 were injected intraperitoneally into mice and then spleen lymphocyte cells were harvested and differentiated cells, M1 (Fig. 5D) and M2 (Fig. 5E), were analyzed by a flow cytometer (Kim et al. 2019). Macrophage M1 cells produces NO or reactive oxygen intermediates to defense bacteria or viruses infection; M2 cells secrete certain cytokines, IL-4 or IL-10, mediating damaged tissues repair (Murray et al. 2014; Rőszer 2015; Kim et al. 2019). Macrophages M1 and M2 polarization were not affected by all pepsin hydrolysates treatments (Fig. 5D, E). Taken together, F1 fraction can increase phagocytosis activities in both mice spleen macrophage and neutrophil cells, but it cannot induce macrophages M1 or M2 polarization.

### Identification of anti-inflammatory peptides by LC–MS/MS from F1 fraction and investigation of synthetic peptide on phagocytosis and polarization of macrophage

**Fig. 6:**
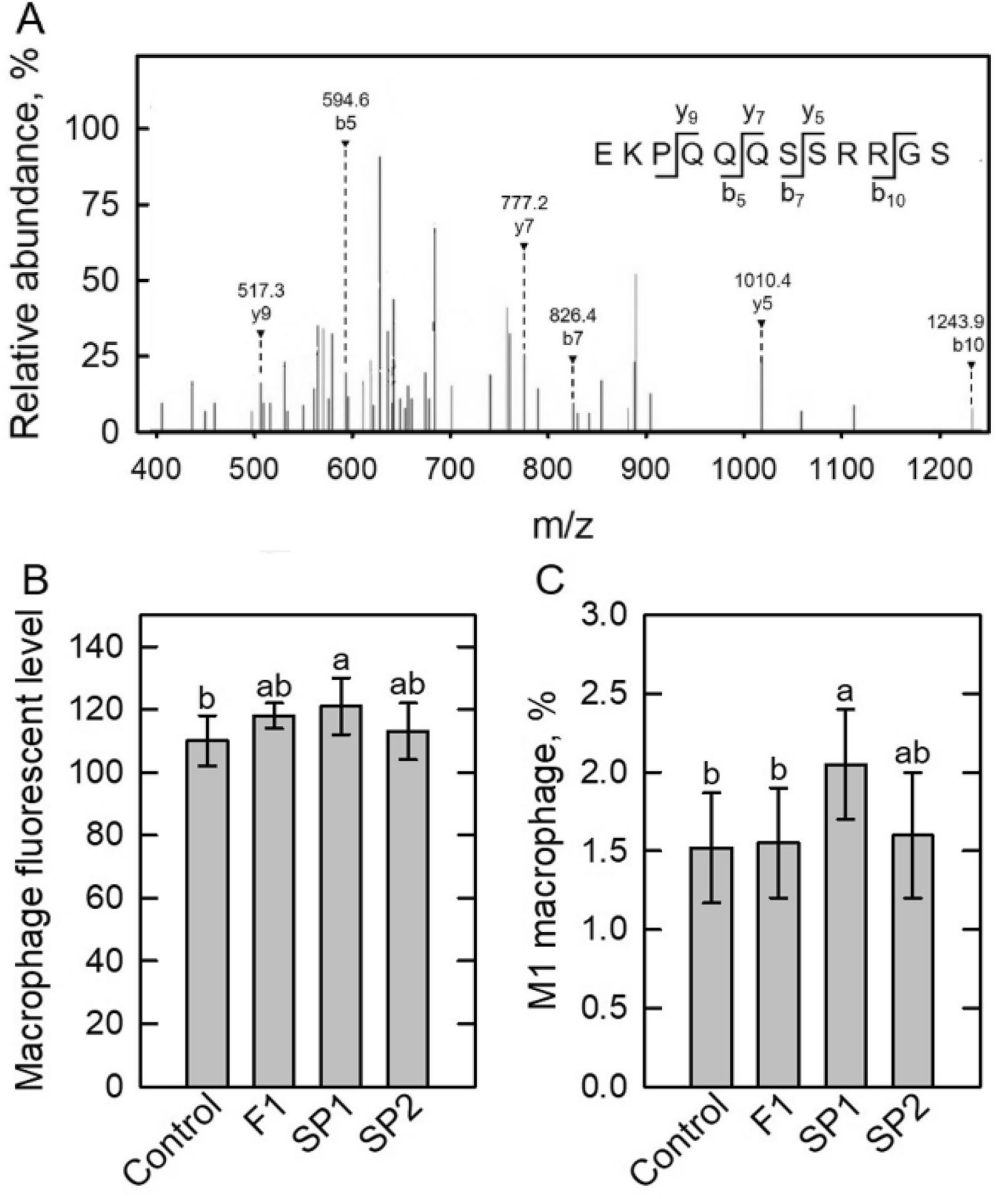
Peptide identified from Pepsin-ISPH4h-1P F1 fraction. **A** the mass spectrum of a peptide, EKPQQQSSRRGS, identified by LC–MS–MS. Effects of the synthetic peptides, EKPQQQSSRRGS (SP1) and VVQGKGAIGFAFP (SP2), on positive fluorescent level in macrophages (**B**), and M1 (**C**) phenotype polarization in mouse spleen in vivo. Bars represent mean ± standard deviation (SD; *n* = 3). Mean with different letters are significantly different (*p* < 0.05) by Duncan’s multiple range test

Previous studies had shown that peptides with positive charged amino acids are intercorrelated with immunomodulatory functions (Mercier et al. 2004; Kong et al. 2008; Jacquot et al. 2010; Hou et al. 2012; Hemshekhar et al. 2019). In this study, liquid chromatography with tandem mass spectrometry (LC–MS–MS) was used for peptide identification (Dia et al. 2014; Fang et al. 2019; Li et al. 2021; Wen et al. 2021). Two peptides with positively charged amino acids, EKPQQQSSRRGS (Fig. 6A) and VVQGKGAIGFAFP, were identified from F1 fraction by LC–MS–MS analysis. Accordingly, synthetic peptide 1 (SP1, EKPQQQSSRRGS) and synthetic peptide 2 (SP2, VVQGKGAIGFAFP) were used to examine its in vivo immunomodulatory effects in mice (Wen et al. 2021). Both synthetic peptides treatments showed no effect on M1 macrophage polarization (data not shown). In macrophage endocytosis analysis, the stimulating effect of the SP1 peptide was better than that of the F1 and SP2 (Fig. 6B). In macrophage polarization analysis, SP1 showed induction effect on M1 macrophage polarization, whereas F1 and SP2 had no effect on that (Fig. 6C). As a result, positive immunomodulatory activities were confirmed in synthetic peptide.

## Conclusions

In this study, pepsin-treated isolated soy protein hydrolysates exhibited immunomodulatory effects such as enhancing phagocytosis activity and not causing excessive inflammatory response. Putative peptides from isolated soy protein hydrolysate by peptic hydrolysis were purified using MWCO and reverse-phase chromatography technique. Two peptides were identified by mass spectrometry. Further studies revealed that the synthetic peptide, EKPQQQSSRRGS, can increase phagocytosis activity in mice spleen macrophage cells as well as can induce macrophages M1 polarization. Taken together, this study can serve as a fundamental basis for the preparation of immunomodulatory peptides from isolated soy proteins.

## Data Availability

Data are contained in the main material.

## Abbreviations

ISPH: Isolated soy protein hydrolysate
MWCO: Molecular weight cut-off
P: Permeate
F: Fraction
LPS: Lipopolysaccharide
IL: Interleukin
TNF-α: Tumor necrosis factor-α
SDS: Sodium dodecyl sulfate
OPA: *O*-Phthalaldehyde
DMSO: Dimethyl sulfoxide
MTT: 3-(4,5-Dimethylthiazol-2-yl)-2,5-diphenyltetrazolium bromide
DMEM: Dulbecco’s modified Eagle medium
FBS: Fetal bovine serum
DH: Degree of hydrolysis
BCRC: Bioresource Collection and Research Center
PBS: Phosphate buffer saline
SD: Standard deviation
HBSS: Hank’s balanced salt solution
GFP: Green fluorescence protein
SP: Synthetic peptide
